# Incremental diagnostic utility of systematic double-bed SPECT/CT for bone scintigraphy in initial staging of cancer patients

**DOI:** 10.1186/s40644-017-0118-4

**Published:** 2017-06-07

**Authors:** Catherine Guezennec, Nathalie Keromnes, Philippe Robin, Ronan Abgral, David Bourhis, Solène Querellou, Romain de Laroche, Alexandra Le Duc-Pennec, Pierre-Yves Salaün, Pierre-Yves Le Roux

**Affiliations:** Service de Médecine Nucléaire, EA3878 (GETBO) IFR 148, CHRU de Brest, Brest, France

**Keywords:** Bone scintigraphy, SPECT/CT, Staging, Cancer, Bone metastasis

## Abstract

**Background:**

SPECT/CT has been shown to increase the diagnostic performance of bone scintigraphy for staging of malignancies. A systematic double-bed SPECT/CT of the trunk may allow further improvement. However, this would be balanced by higher dosimetry and longer acquisition time. The objective was to assess the incremental diagnostic utility of a systematic double-bed SPECT/CT acquisition for bone scintigraphy in initial staging of cancer patients, especially compared with the usual approach consisting in a whole body planar scan (WBS) plus one single-bed targeted SPECT/CT.

**Methods:**

One hundred two consecutive patients referred for bone scintigraphy for initial staging of malignancy were analyzed. All patients underwent a double-bed SPECT/CT acquisition of the trunk. Images were interpreted by two nuclear medicine physicians in a 3-step procedure. Firstly, only WBS planar images were used; secondly, one additional single-bed SPECT/CT chosen based on planar images was used; finally, WBS planar and double-bed SPECT/CT images were interpreted. Lesions were classified as benign, equivocal or suspicious for metastasis. A per-lesion, per-anatomical region and per-patient analysis was performed.

**Results:**

In a per-lesion analysis, the number of equivocal and suspicious lesions was 91 and 241 using WBS planar images, 17 and 259 using a single-bed SPECT/CT acquisition and 11 and 269 using double-bed SPECT/CT images, respectively. In a per-patient analysis, the diagnostic conclusion was negative, equivocal or suspicious for malignancy in 35, 53 and 14 patients using WB planar images, 77, 6 and 19 patients using an additional single-bed SPECT/CT and 76, 7 and 19 using double-bed SPECT/CT images, respectively.

Seventeen lesions unseen on WBS images were interpreted as suspicious (*n* = 12) or equivocal (*n* = 5) on double-bed SPECT/CT images. Six lesions unseen on “WBS + targeted single-bed SPECT/CT” were interpreted as suspicious on double-bed SPECT/CT, with no shift in the metastatic status of patients.

**Conclusion:**

A systematic double-bed SPECT/CT acquisition has a limited incremental diagnostic value over an oriented single-bed SPECT/CT in terms of specificity and conclusiveness of bone scintigraphy in the initial staging of cancer patients. However, it slightly improved the sensitivity of the test by detecting unseen lesions on WBS, which may be of value for initial staging of cancer.

## Background

Evaluating the metastatic status in cancer is of utmost importance in order to provide the best patient’s management. Bone scintigraphy is currently a reference test in the initial staging of cancer, mainly prostate and breast cancers, to assess the presence of metastatic lesions. The accuracy of staging is a major challenge since the whole patient management may completely change, from a curative and local treatment for local diseases to a palliative treatment for metastatic patients in most cases [1–3].

Bone scintigraphy historically consists in a planar whole-body acquisition (whole-body scintigraphy -WBS), which is quickly acquired and has a large field of view. WBS has been proved to have a high sensitivity in detecting metastasis. However, the tracer uptake not being tumor-specific, its specificity is quite low [4–7]. Technologic innovation of these past 20 years has offered the opportunity to perform SPECT (single photon-emission computed tomography), then SPECT/CT, in addition to the WBS in clinical routine. SPECT has been shown to be more accurate than WBS to distinguish between malignant and benign lesions [8, 9]. SPECT/CT allows an even better characterization of equivocal uptakes on WBS by differentiating metastatic from benign lesions such as degenerative changes, fractures or other benign lesions [10, 11]. As a result, SPECT/CT has been shown to dramatically reduce the proportion of inconclusive results and increase the specificity of bone scintigraphy [5, 12–14]. Therefore, in most of nuclear medicine centers, the usual protocol for staging of bone metastases consists in a whole-body planar acquisition followed, if needed, by a targeted SPECT/CT to characterize suspicious or equivocal uptakes seen on WBS.

There is much less data on the usefulness of SPECT/CT to improve the sensitivity of the test. Some studies reported a slight increase of sensitivity which would be of interest in the setting of initial staging of malignancy [15]. Accordingly, although increasing the acquisition time and the effective dose, a systematic double-bed SPECT/CT of the trunk may be proposed to improve both specificity and conclusiveness but also sensitivity of bone scan in the initial staging of malignancy. Some studies have shown the diagnostic utility of a double-bed SPECT/CT as compared with WBS alone [15]. However, no study has yet compared the diagnostic performance of a systematic double-bed SPECT/CT of the trunk compared to the commonly used “WBS plus one single–bed targeted SPECT/CT” strategy.

The aim of this study was to assess the incremental diagnostic utility of a systematic double-bed SPECT/CT acquisition for bone scintigraphy in initial staging of cancer patients compared with the conventional “WBS plus single-bed targeted SPECT/CT” strategy.

## Methods

### Patients

Consecutive patients referred for bone scintigraphy for initial staging of biopsy proven malignancy to the nuclear medicine department of Brest University Hospital, France, from February to June 2014, were analyzed. Exclusion criteria included monoclonal gammapathy, patients under 18 years of age, technical issues not allowing a double–bed SPECT/CT acquisition, double-bed SPECT/CT acquisition not centered from the upper cervical spine to the proximal femora. The study was performed in accordance with the Declaration of Helsinki and was approved by the institutional ethics committee (Number 2015, CE26). All patients gave their informed consent.

### Image acquisition

Bone scintigraphy systematically consisted in a planar whole-body scintigraphy (WBS) and a double-bed SPECT/CT from the cervical spine to the proximal femora. Images were acquired on Symbia Intevo 6 and Symbia T6 gamma-cameras (Siemens Healthcare, Erlangen, Germany). Both these hybrid systems incorporate a 6-slice X-ray CT scanner, and allow the acquisition of coregistered CT and SPECT images in one session. The acquisition was standard with low-energy high-resolution (LEHR) collimators, energy window 140 keV (+/- 7,5%), WBS was performed approximately 3 h after the intravenous injection of 9 MBq/kg of 99mTc-DPD (99mTc 3,3-diphosphono-1,2-propanedicarboxylic acid - Teceos®, IBA Molecular, Gif-sur-Yvette, France). Planar images were acquired with the following parameters: image matrix 256 × 1024, scanning speed 32 cm/min post-filtered with OncoFlash (Siemens Medical Solutions, USA). A double-bed SPECT/CT was acquired immediately after WBS from the upper cervical spine to the proximal femora. SPECT images were obtained with the following parameters: 10 secondes per step acquiring 120 projections with 180° rotation for each camera head, on a 128 × 128 pixel matrix. SPECT data were reconstructed using Flash 3D (Siemens) with ordered subset expectation maximization (OSEM) (8 iterations, 16 subsets and 10 mm Gaussian post filtering). CT imaging consisted in a low-dose technique with the following parameters: modulated tube current intensity (Care4D, 90mAs), 130 kV, total collimation 6x1mm, pitch 1, and was performed on the same anatomical region as the SPECT. The estimated irradiation dose received by the patients was simulated with the CT-Expo v2.1 package.

### Image interpretation

Images were interpreted by two nuclear medicine physicians in a 3-step procedure and by consensus. Firstly, only WBS planar images were considered. Secondly, a single-bed SPECT/CT chosen based on planar images was used if WBS demonstrated any equivocal or suspicious uptake. Finally, WBS and double-bed SPECT/CT images were used for interpretation. A per-lesion, a per-anatomical region and a per-patient analysis were performed. Ten different regions were considered: cervical spine, thoracic spine, lumbar spine, pelvis, ribs, sternum, shoulders, skull, femora, other [15]. Each lesion was registered up to a maximum of 10 lesions per anatomical region. At each step, lesions, regions and diagnostic conclusions were classified using a 3-level scale, as negative for malignancy, equivocal or suspicious for metastasis [14].

## Results

### Patients’ characteristics

Between February and June 2014, 104 consecutive patients referred for initial staging of malignancy underwent a planar whole body scintigraphy and a double-bed SPECT/CT of the trunk. Two patients could not be analyzed due to technical problems (one CT and one CT + SPECT lacking in the PACS system). One hundred and two patients were analyzed (male = 79, female = 23, mean age +/- SD = 68,7 +/- 11,5 years). The repartition of cancer was as follows: prostate *n* = 67, breast *n* = 17, lung *n* = 6, bladder *n* = 6, kidney *n* = 4, brain *n* = 1, ovary *n* = 1. The estimated effective dose received by the patients was 10,2 mSv with the double-bed SPECT/CT.

### Whole body scintigraphy

**Fig. 1:**
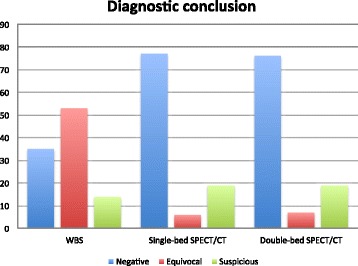
Diagnostic conclusion

Results of WBS interpretation are displayed in Table 1 and Fig. 1. Distribution of suspicious and equivocal regions is shown in Table 2. On WBS planar images, the number of equivocal and suspicious lesions was 91 and 250, respectively. The diagnostic conclusion was negative, equivocal or suspicious for malignancy in 35 (34.3%), 53 (52%) and 14 (13.7%) patients, respectively.

**Table 1:** Number and status of lesions, regions, and diagnostic conclusion for each modality

	WBS	Single-bed SPECT/CT	Double-bed SPECT/CT
Lesions			
Suspicious	250	262	265
Equivocal	91	17	18
Regions			
Suspicious	58	64	70
Equivocal	67	10	9
Benign	997	1048	1043
Diagnostic conclusion			
Suspicious	14	19	19
Equivocal	53	6	7
Negative	35	77	76

**Table 2:** Distribution of suspicious and equivocal regions between anatomical regions

	WBS			Single SPECT/CT			Double-bed SPECT/CT		
Regions	S	E	B	S	E	B	S	E	B
Cervical spine	3	1	98	3	1	98	5	0	97
Thoracic spine	7	11	84	9	2	91	10	1	91
Lumbar spine	7	16	79	8	1	93	8	1	93
Pelvis	9	18	75	8	2	92	10	2	90
Ribs	9	11	82	11	2	89	13	1	88
Sternum	4	2	96	4	0	98	3	1	98
Shoulders	4	2	96	6	0	96	6	1	95
Skull	5	0	97	5	0	97	5	0	97
Femora	6	5	91	7	1	94	7	1	94
Others	4	1	97	3	1	98	3	1	98

*S* suspicious, *E* equivocal, *B* benign

### WBS + targeted single-bed SPECT/CT strategy

With this strategy, the number of equivocal and suspicious lesions was 17 and 262, respectively; the diagnostic conclusion was negative, equivocal or suspicious for malignancy in 77 (75.5%), 6 (5.9%) and 19 (18.6%) patients, respectively (See Table 1 and Fig. 1).

Comparison between WBS and WBS + one single-bed SPECT/CT interpretations is shown in Table 3. Out of the 91 equivocal uptakes on WBS images, 65 (71.4%) were classified as benign and 13 (14.3%) as suspicious, while only 13 (14.3%) remained equivocal using one additional single-bed SPECT/CT acquisition. Similarly, out of the 53 equivocal diagnostic conclusions on WBS, 39 (73.6%) were re-classified as negative and 8 (15.1%) as suspicious.

**Table 3:** Comparison between WBS and single-bed SPECT/CT

	Single-bed SPECT/CT		
WBS	Suspicious	Equivocal	Benign
Lesions			
Suspicious	243	0	7
Equivocal	13	13	65
Benign	6	4	
Regions			
Suspicious	53	0	5
Equivocal	9	9	49
Benign	2	1	994
Diagnostic conclusion			
Suspicious	11	0	3
Equivocal	8	6	39
Negative	0	0	35

Six lesions unseen on WBS images were interpreted as suspicious on SPECT/CT images. Four additional lesions unseen on WBS were classified as equivocal on SPECT/CT acquisition. None of these 10 new lesions induced a shift in the metastatic status of patients.

### WBS + systematic double-bed SPECT/CT strategy

With this strategy, the number of equivocal and suspicious lesions was 18 and 265 respectively; the diagnostic conclusion was negative, equivocal or suspicious for malignancy in 76 (74.5%), 7 (6.9%) and 19 (18.6%) patients, respectively (See Table 1 and Fig. 1).

#### Comparison with WBS

Comparison between WBS and WBS + systematic double-bed SPECT/CT interpretation is shown in Table 4. Out of the 91 equivocal uptakes on WBS images, 65 (71.4%) were re-classified as benign and 18 (19.8%) as suspicious, while only 8 (8.8%) remained equivocal using a double-bed SPECT/CT acquisition. Similarly, out of the 53 equivocal diagnostic conclusions, 38 (71.7%) were re-classified as negative and 8 (15.1%) as suspicious.

**Table 4:** Comparison between WBS and double-bed SPECT/CT

	Double-bed SPECT/CT		
WBS	Suspicious	Equivocal	Benign
Lesions			
Suspicious	235	5	10
Equivocal	18	8	65
Benign	12	5	
Regions			
Suspicious	51	2	5
Equivocal	13	5	49
Benign	6	2	989
Diagnostic conclusion			
Suspicious	11	0	3
Equivocal	8	7	38
Negative	0	0	35

Twelve lesions unseen on WBS images were interpreted as suspicious on double-bed SPECT/CT images. Five additional lesions unseen on WBS were classified as equivocal on double-bed SPECT/CT acquisition.

#### Comparison with WBS + single-bed SPECT/CT strategy

Comparison between “WBS + single-bed SPECT/CT” and “WBS + double-bed SPECT/CT” interpretation is shown in Table 5. Out of the 17 equivocal uptakes on WBS + single-bed SPECT/CT images, none was re-classified as benign, 5 (29.4%) as suspicious, and 12 (70.6%) remained equivocal using a double-bed SPECT/CT acquisition. These lesions were found on patients already considered suspicious on both modalities. Out of the 262 suspicious uptakes on WBS + single-bed SPECT/CT, 5 (1.9%) were re-classified as equivocal and 3 (1.1%) as benign, while 254 (97%) remained suspicious.

**Table 5:** Comparison between single-bed SPECT/CT and double-bed SPECT/CT

	Double-bed SPECT/CT		
Single-bed SPECT/CT	Suspicious	Equivocal	Benign
Lesions			
Suspicious	254	5	3
Equivocal	5	12	0
Benign	6	1	
Regions			
Suspicious	62	2	0
Equivocal	4	6	0
Benign	4	1	1043
Diagnostic conclusion			
Suspicious	19	0	0
Equivocal	0	6	0
Negative	0	1	76

Six lesions unseen by the “WBS + one single-bed SPECT/CT” strategy were interpreted as suspicious on double-bed SPECT/CT images, corresponding to 4 new suspicious regions. These new lesions did not induce a shift in the metastatic status of patients. The 4 suspicious regions concerned 3 (3%) patients. Two patients had lung cancers with diffuse bone metastases. The third one had prostate cancer with only one suspicious region (lumbar spine) on single-bed SPECT/CT and two more suspicious regions (ribs and shoulders) on double-bed SPECT/CT.

**Fig. 2:**
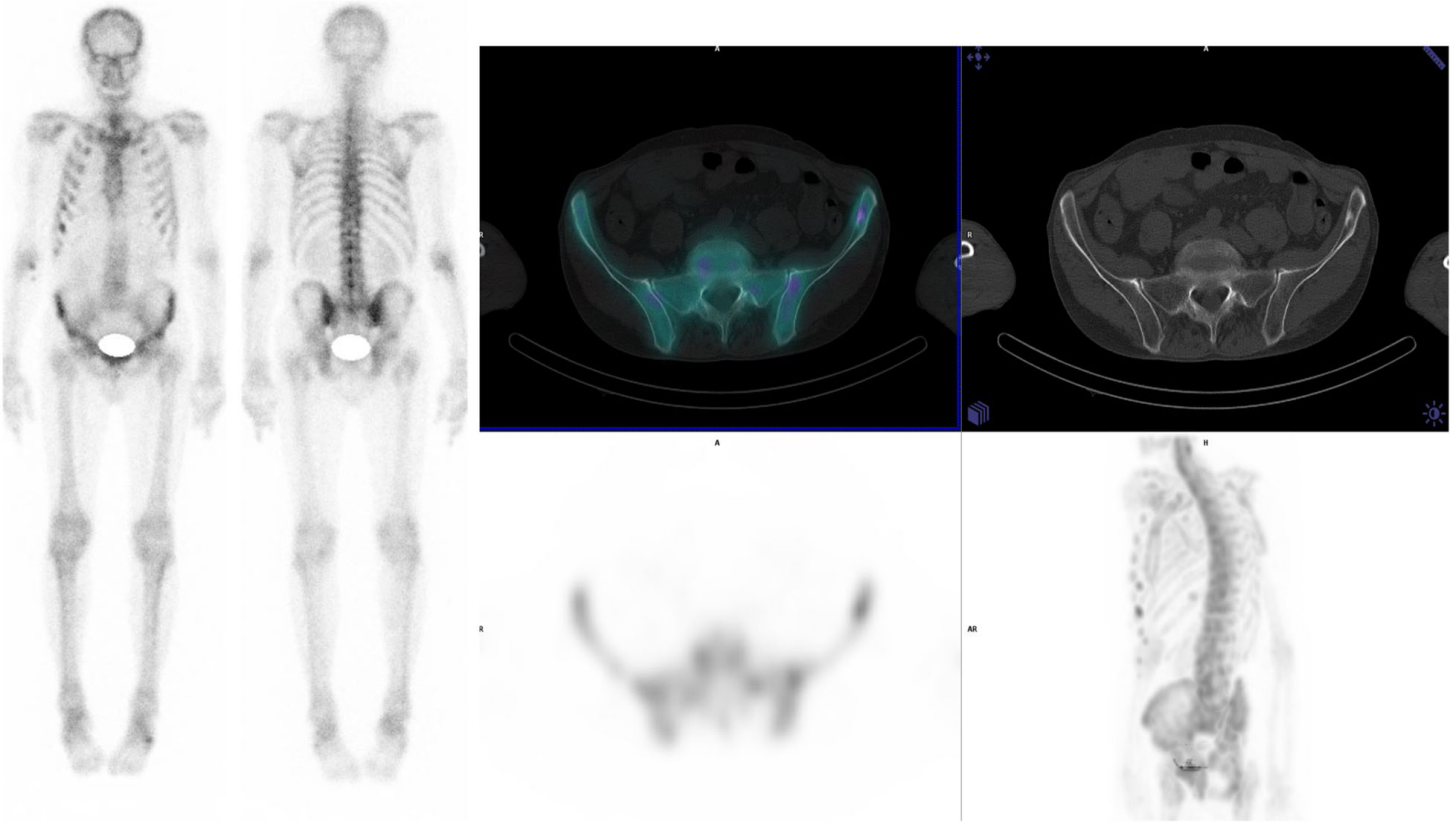
Equivocal sclerotic lesion on the left iliac aisle found on double-bed SPECT/CT, inducing an equivocal diagnostic conclusion. The patient had prostate cancer. A guided biopsy did not show malignancy

One additional lesion unseen on single-bed SPECT/CT was classified as equivocal on double-bed SPECT/CT acquisition. It induced a change in conclusion diagnostic in one patient with prostate cancer (1%) from benign to equivocal. WBS was interpreted as equivocal, with 2 equivocal lesions on the ribs. Based on single-bed SPECT/CT on the thorax, the 2 lesions on the ribs were re-classified as benign. On the double-bed SPECT/CT, 1 equivocal lesion was found in the iliac bone on the scan inducing an equivocal diagnostic conclusion. A guided biopsy of the equivocal lesion did not show malignancy (See Fig. 2). No patient had a change of diagnostic conclusion to suspicious for malignancy on double-bed SPECT/CT.

## Discussion

We evaluated in this study the incremental diagnostic utility of a systematic double-bed SPECT/CT acquisition for the initial staging of cancer patients with bone scintigraphy. Consistent with previous data, adding SPECT/CT to WBS drastically reduced the number of equivocal lesions and diagnostic conclusions in favor of a higher specificity. However, comparing systematic double-bed SPECT/CT with single-bed SPECT/CT strategies, the incremental value was limited in terms of specificity and conclusiveness. On the other hand, a systematic double-bed SPECT/CT increased the sensitivity of bone scan, identifying unseen lesions and reclassifying regions considered benign on the WBS as suspicious on SPECT/CT, with a potential therapeutic impact on patient management.

Adding SPECT/CT to WBS drastically reduced the number of equivocal diagnostic conclusion in favor of a higher specificity of SPECT/CT compared to WBS alone. Indeed, out of the 53 patients with an “equivocal” diagnostic conclusion on WBS, only 7 patients’ staging remained “equivocal” with double-bed SPECT/CT. In per-lesion analysis, 71.4% and 19.8% of equivocal uptake on WBS images were reclassified as benign and suspicious, respectively, while only 8.8% remained equivocal. This data is consistent with previous studies [5, 12–14]. In a study from Palmedo et al., 47.3%, 18.7% and 34% of lesions were considered benign, equivocal and suspicious on WBS, and 60.1%, 3.5% and 36.4% with a double-bed SPECT/CT, also showing a higher shift from equivocal to benign than to suspicious lesions [15]. In another publication from Heylar et al., out of equivocal lesions seen on WBS, 68% were reclassified as benign, 24% as suspicious and 8% remained equivocal [16]. Our data confirms the increase in specificity when adding SPECT/CT to WBS.

In terms of specificity and conclusiveness, the impact of a systematic double-bed SPECT/CT acquisition compared to a single-bed SPECT/CT acquisition was limited. Indeed, there was no change in the proportion of equivocal and suspicious lesions (6.4% and 6.1%, respectively) or regions (93.6% and 93.9%, respectively). Nevertheless, the impact was not trivial as 5 equivocal lesions were re-classified as suspicious and conversely 5 suspicious lesions were downstaged as equivocal on double-bed SPECT/CT.

The ability of SPECT/CT to improve bone scan sensitivity is more controversial. Palmedo et al. reported in a large series a slight impact in breast cancer but not in prostate cancer [15]. In our study, we found an increase in sensitivity when adding a double-bed SPECT/CT. Overall, SPECT/CT detected 12 suspicious and 5 equivocal lesions unseen on WBS in 5 and 3 patients, respectively. Moreover there was also a slight increase in sensitivity when comparing WBS + single-bed SPECT/CT with WBS + double-bed SPECT/CT. Indeed, double-bed SPECT/CT detected 6 suspicious and 1 equivocal lesions in 4 and 1 patients respectively, when compared with single-bed SPECT/CT. The 6 suspicious lesions concerned 4 new suspicious regions in 3 (3%) patients. This increased sensitivity appears relevant in the setting of initial staging of cancer.

In our series, the impact on patient’s management was however limited. There was only one change in diagnostic conclusion using the double-bed SPECT strategy as compared with the single-bed SPECT strategy. No patient was upstaged to suspicious for metastasis. The only change in diagnostic conclusion, from benign to equivocal, concerned a patient with prostate cancer. On double-bed SPECT/CT, there was a slight uptake in the iliac bone on an equivocal morphologic lesion. However, a guided biopsy did not show malignancy (Fig. 2). Three patients had the same diagnostic conclusion (evidence of bone metastases) but had additional lesions on double-bed SPECT/CT images not seen on WBS. Out of them, 2 had lung cancers with diffuse bone metastases, with no therapeutic consequences. The third one had prostate cancer with one isolated suspicious region (lumbar spine) on single-bed SPECT/CT and two additional suspicious regions (ribs and shoulders) on double-bed SPECT/CT. In this patient, this increase in sensitivity could potentially have a therapeutic impact, especially with the development of radiotherapy with curative aim for oligometastatic disease [17]. Oligometastatic disease concerns patients with 1 to 5 suspicious lesions [18]. In our study, we found no shift from oligometastatic to multi-metastatic status or from multi-metastatic to oligometatastic status. Indeed, amongst the patients with an oligometastatic status, when analyzing new suspicious lesions on double-bed SPECT/CT, 4 patients initially oligometastatic with respectively 1, 1, 1 and 4 suspicious lesions remained oligometastatic on double-bed SPECT/CT with respectively 3 (2 new suspicious lesions overlooked on WBS + single-bed SPECT/CT), 2, 2 and 5 (1 equivocal lesion becoming suspicious for these last 3 patients). On the other hand, when analyzing the lesion shifts from suspicious to equivocal or benign, they all concerned the same patient who had prostate cancer with diffuse bone metastases. However, depending on the location of the metastasis or the symptoms associated with them, the increased sensitivity may also support a palliative treatment such as analgesic radiotherapy. Moreover this more precise characterization of the number and location of suspicious lesions on double-bed SPECT/CT may also better evaluate the therapeutic response to chemotherapy, hormonotherapy or internal radiotherapy treatment.

In terms of radiation exposure, a systematic double SPECT/CT acquisition induces an approximately 5 mSv increase of the effective dose. Indeed the estimated effective dose received by the patients was 10,2 mSv for a double-bed SPECT/CT versus 4,7 mSv for a single-bed SPECT/CT (abdomen). In addition, a systematic double-bed SPECT/CT is approximately 13 min longer compared to a single-bed SPECT/CT. These inconveniences appear acceptable in the setting of staging of cancer, if a double-bed SPECT/CT increases the sensitivity of the test and prevents further other irradiating examinations to specify undetermined lesions.

In these past 10 years, instrumentation of gamma cameras has evolved a lot, in terms of physics properties and reconstruction methods, resulting in an improved sensitivity. In our study, we used Flash 3D reconstruction method. In parallel, with the development of PET/CT, 18-F FNa PET/CT has been adopted in some centres as an alternative to bone scintigraphy in the detection of bone metastases, with a high sensitivity and specificity, and was showed to outperform SPECT/CT in several studies [5].

There are limitations in our study that deserve further discussion. Firstly, we included consecutive patients referred for initial staging of cancer whatever the type of cancer. The impact of a double-bed SPECT/CT may be different according to the primary. Nevertheless, this approach reflects the usual activity of a nuclear medicine department proposing the same protocol for all cancer patients. Secondly, the scale of our study was limited with a small number of patients. Larger studies including more patients would help further analyzing the impact on diagnostic conclusion of a systematic double SPECT/CT with an inter-observer reproducibility analysis. Thirdly, the targeted SPECT/CT strategy could also consist in a targeted double-bed SPECT/CT when needed, depending on the lesions seen on WBS. However, in our study, the main interest of a systematic double-bed SPECT/CT was to detect unseen lesions of WBS. Acquiring single or double SPECT/CT acquisitions on the base of WBS interpretation would not increase the sensitivity of the test. Moreover, some studies proposed a multi-bed SPECT/CT [19]. In our study, the double-bed SPECT/CT was only performed from the cervical spine to proximal femora, and did not include the lower limbs and the skull. However, metastatic lesions of extremities were previously found to be very rare without an axial extension [15, 20, 21]. Finally, in our study, the scanning speed when acquiring WBS was 32 cm/min, quite fast when compared with previous studies. Yet, we used a post-treatment denoising step using a Pixon method, Oncoflash. This method produces an image equivalent to the one deriving from an acquisition half as fast, thus in our case at a scanning speed of 16 cm/min, which is average when compared with other studies [22].

## Conclusions

A systematic double-bed SPECT/CT acquisition has a limited incremental diagnostic value over an oriented single-bed SPECT/CT in terms of specificity and conclusiveness of bone scintigraphy in the initial staging of cancer patients. However, it slightly improves the sensitivity of the test by detecting unseen lesions on WBS, which may be of value for initial staging of cancer.

## Abbreviations

SPECT/CT: Single-Photon Emission Computed Tomography/Computed Tomography
WBS: Whole-body scintigraphy
